# TBHQ attenuates ferroptosis against 5-fluorouracil-induced intestinal epithelial cell injury and intestinal mucositis via activation of Nrf2

**DOI:** 10.1186/s11658-021-00294-5

**Published:** 2021-11-18

**Authors:** Shihua Deng, Dongming Wu, Li Li, Jin Li, Ying Xu

**Affiliations:** 1 School of Clinical Medicine, Chengdu Medical College, Chengdu, Sichuan 610500 People’s Republic of China; 2 The First Affiliated Hospital of Chengdu Medical College, Chengdu, Sichuan 610500 People’s Republic of China; 3 School of Laboratory Medicine, Chengdu Medical College, Chengdu, Sichuan 610500 People’s Republic of China

**Keywords:** Tertiary butylhydroquinone, Intestinal mucositis, Ferroptosis, 5-fluorouracil

## Abstract

**Background:**

Intestinal mucositis is a common side effect of chemotherapy and radiotherapy. Very few drugs can efficiently ameliorate it. Tertiary butylhydroquinone (TBHQ) is a widely used food preservative with known immunomodulatory activity. Whether it has an effect on intestinal mucositis remains unknown. In this study, we investigated the role and mechanism of action of TBHQ on 5-fluorouracil-induced (5-FU-induced) human intestinal epithelial cell (HIEC) injury and intestinal mucositis in mice.

**Methods:**

We established a cell model of HIEC injury and a mouse model of intestinal mucositis via treatment with 5-FU. Cell death, Cell Counting Kit-8, and lactate dehydrogenase (LDH) release were assessed for the HIECs. Diarrhea, body weight, intestinal length, mucosal damage, and the levels of IL-6, TNF-α, IL-1β, glutathione, reactive oxygen species, and malondialdehyde were determined for the mice. Additionally, we performed immunohistochemical analysis, immunofluorescence, western blotting, quantitative real-time PCR, and ELISA to examine the effects of TBHQ. Finally, HIECs were transfected with an Nrf2 gene silencer to verify its role in ferroptosis. All data were analyzed using one-way analysis of variance or paired t-tests.

**Results:**

TBHQ markedly decreased LDH release and cell death and improved the proliferative ability of 5-FU-treated HIECs*.* The TBHQ-treated mice showed reduced weight loss, a lower diarrhea score, and longer colons than the 5-FU-treated mice. The in vivo expressions of IL-1β, IL-6, and TNF-α were suppressed by TBHQ treatment. Ferroptosis was shown to be involved in 5-FU-induced intestinal mucositis, and TBHQ markedly hampered its activation. Mechanistically, TBHQ activated Nrf2 effectively and selective Nrf2 knockdown significantly reduced the anti-ferroptotic functions of TBHQ in 5-FU-treated HIECs.

**Conclusions:**

TBHQ attenuates ferroptosis in 5-FU-induced intestinal mucositis, making it a potential novel protective agent against intestinal mucositis.

**Supplementary Information:**

The online version contains supplementary material available at 10.1186/s11658-021-00294-5.

## Background

A major adverse effect of chemotherapy drugs is their impact on the intestinal mucosa. They kill rapidly differentiating intestinal mucosal cells, leading to a type of injury called intestinal mucositis [1, 2]. Symptoms include diarrhea, hematochezia, and anorexia. The incidence of intestinal mucositis caused by chemotherapy is approximately 40%, with 90% of cases involving 5-fluorouracil (5-FU) and methotrexate [2].

5-FU is a chemotherapeutic drug that is widely used in the treatment of colon, stomach, esophagus, and other digestive system tumors [3, 4]. Very few drugs can ameliorate 5-FU-induced intestinal mucositis, making the development of effective drugs highly important.

Ferroptosis is a non-apoptotic form of cell death. It is defined as an iron-dependent regulatory necrosis that is caused by a large amount of lipid peroxidation-mediated membrane damage [5]. It may play a key role in the occurrence and development of various diseases [6–9]. Several studies have shown that it occurs in inflammatory diseases and that its inhibition is effective in alleviating these conditions. For example, Li et al. [10] reported that ferrostatin-1 (Fer-1), a ferroptosis inhibitor, alleviates angiotensin II-induced inflammation and ferroptosis by suppressing reactive oxygen species (ROS) formation and activating the Nrf2/HO-1 signaling pathway. Cao et al. [11] demonstrated that the ferroptosis inhibitor liproxstatin-1 attenuates neuroinflammation after subarachnoid hemorrhage. Xu et al. [12] found that ferroptosis is involved in intestinal epithelial cell death in ulcerative colitis and suggested it as a potential therapeutic target for that disease.

It is clear from these studies that ferroptosis involves multiple types of tissue injury and inflammation. However, its role in chemotherapy-related intestinal mucositis has not yet been elucidated.

Nuclear factor erythroid 2-related factor 2 (Nrf2) is a transcription factor mainly activated by cellular oxidative stress [13]. It plays a vital role in cellular antioxidant responses. Ferroptosis is mainly caused by lipid peroxidation, which is closely related to oxidative stress [5]. Previous studies have shown that activation of the Nrf2 pathway can significantly inhibit ferroptosis in various diseases [10, 14–16].

Tertiary butylhydroquinone (TBHQ), an activator of the Nrf2 signaling pathway, is a commonly used food antioxidant that is widely found in oils, biscuits, and other foods [17]. Researchers have demonstrated that TBHQ induces remarkable antioxidant activity in multiple types of cells and tissues by activating Nrf2 [17–19]. In this study, we aimed to investigate whether TBHQ could inhibit ferroptosis and attenuate 5-FU-induced intestinal mucositis by activating Nrf2.

## Methods

### Reagents

Antibodies against Nrf2 (16396–1-AP), HO-1 (66743–1-Ig), ZO-1 (21773–1-AP), occludin (13409–1-AP), GAPDH (60004–1-Ig), and HRP-conjugated secondary antibodies (SA00001-1 and SA00001-2) were purchased from Proteintech (China). Antibodies against claudin-5 (343214) were purchased from Zenbio (China). Antibodies against GPX4 (ab125066) and 4HNE (ab46545) were obtained from Abcam (UK). 5-FU, TBHQ, and Fer-1 were purchased from Selleck Chemicals (USA).

### Cell cultures and transfections

Cells of the human intestinal epithelial cell (HIEC) line were obtained from the Cell Bank of the Chinese Academy of Sciences (China). They were grown at 37 °C in 5% CO_2_. The Nrf2-silencing lentivirus and control lentivirus were packaged by Genomeditech (Shanghai, China). The Nrf2-silencing targeting sequences were: shControl, 5′-TTCTCCGAACGTGTCACGT-3′, and shNrf2, 5′-GTCCAAAGAGCAGTTCAATGA-3′. Cell transduction was performed according to the manufacturer’s instructions. Stable cells were selected using puromycin.

### Cell death assay

For the cell death assay, HIECs were collected, stained with 7-aminoactinomycin D (7-AAD; 2 μg/ml in PBS, KeyGEN, China) for 20 min, and washed three times. A portion of the cells was then analyzed using a flow cytometer (FACSCalibur, Becton–Dickinson, USA). The other portion was permeated using 0.5% Triton X-100 (Sigma-Aldrich, USA) for 2 min and washed three times. Thereafter, the nuclei were stained with DAPI and then washed three times. The number of 7-ADD positive cells was counted under a fluorescence microscope (DM4000B, Leica, Germany).

### Cell Counting Kit-8 assay

HIECs (5000 cells/well) were seeded onto 96-well plates. Next, the cells were treated under the conditions indicated in figure lables. After 24 or 48 h, the relative number of viable cells was determined by incubating the cells with the reagents supplied in the Cell Counting Kit-8 (CCK-8; Selleck). The optical density of the microplate wells was recorded at 450 nm.

### Lactate dehydrogenase (LDH) release assay

HIECs (5000 cells/well) were seeded onto a 96-well plate and incubated overnight at 37 °C in a cell incubator containing 5% CO_2_. After receiving the corresponding treatment in figure lables, the LDH release assay was performed using the LDH Cytotoxicity Assay Kit (Beyotime, China), according to the manufacturer’s instructions.

### Animals and experimental design

All the experimental protocols were approved by the Laboratory Animal Ethical Committee of Chengdu Medical College. Male C57/BL6 mice (eight weeks old, 18–20 g) were purchased from Chengdu Dossy Experimental Animals (China).

All the mice were housed in plastic cages with free access to food and water at 25 °C with a 12 h light/dark cycle. The mice were randomly divided into four groups (n = 6 mice/group): the control group, 5-FU group, 5-FU + TBHQ group, and 5-FU + Fer-1 group. 50 mg/kg body weight 5-FU was intraperitoneally (i.p.) injected into the mice of the 5-FU, 5-FU + TBHQ and 5-FU + Fer-1 group per day for five days to induce intestinal mucositis. Starting on the same day, the mice in the 5-FU + TBHQ and 5-FU + Fer-1 group were treated with TBHQ (10 mg/kg body weight; in DMSO, i.p. injection) or Fer-1 (2.5 μmol/kg body weight; in DMSO; i.p. injection) once daily for eight days (days 1–8). Starting on the same day, the mice of the 5-FU group were treated with equivalent volumes of dimethyl sulfoxide (DMSO) for eight days.

Body weight and diarrhea assessments were conducted daily. On day 9, the animals were killed. Intestine lengths were measured and histopathological analyses were performed for all the animals.

### ELISA

Mouse small intestines were collected and the levels of IL-6, IL-1β, and TNF-α were detected using ELISA kits (Mlbio, China) according to the manufacturer's protocol.

### Quantitative real-time PCR

Quantitative real-time PCR was performed as previously described [20]. β-actin was validated as an internal control. The following primers were used: Nrf2-forward, 5′-TAAAGCACAGCCAGCACATTCTCC-3′ and reverse, 5′-TGATGACCAGGACTCACGGGAAC-3′; β-actin-forward, 5′-CCTGGCACCCAGCACAAT-3′ and reverse, 5′-GGGCCGGACTCG TCATAC-3′.

### Immunohistochemical analysis (IHC), immunofluorescence (IF), western blotting, and hematoxylin and eosin (H&E) staining

IHC, IF, and western blotting assays were performed to measure the expressions of 4-HNE, GPX4, Nrf2, HO-1, ZO-1, occludin and claudin-5. H&E staining was performed to measure the pathological change in intestinal tissue using our previously described method [21].

### Measurement of glutathione (GSH) and lipid peroxidation levels

The GSH levels in the HIECs and intestinal tissues were measured using a GSH assay kit (Beyotime Biotechnology, China). The degree of lipid peroxidation in the HIECs and intestinal tissues was analyzed by measuring malondialdehyde (MDA) levels using a lipid peroxidation assay kit (Beyotime Biotechnology).

### Quantitation of ROS

Dihydroethidium (DHE; Molecular Probes, USA) staining was used to determine ROS levels in the HIECs and intestinal tissues. The sections were dewaxed, dehydrated with an ethanol gradient, and, after washing with PBS, tissue sections or HIECs were stained with 5 mmol/l DHE (in PBS) for 20 min at 25 °C. The nuclei were stained with DAPI. Finally, fluorescence images of the intestinal tissue sections or HIECs were randomly captured at 200 × magnification using a fluorescence microscope. The fluorescence intensity was analyzed using Image J software. Using the ROS Assay kit (Beyotime, China), the ROS level was measured via flow cytometry (Fig. 5K), according to the manufacturer's protocol.

### Statistical analysis

Each in vitro experiment was performed independently at least three times. All animals were randomly assigned to experimental groups. Statistical significance among the groups was determined using one-way analysis of variance or paired t-tests. All the statistical analyses were performed using GraphPad Prism 5 (GraphPad Software, USA). Statistical significance was set at p < 0.05.

## Results

### TBHQ alleviates 5-FU-induced intestinal epithelial cell injury

**Fig. 1:**
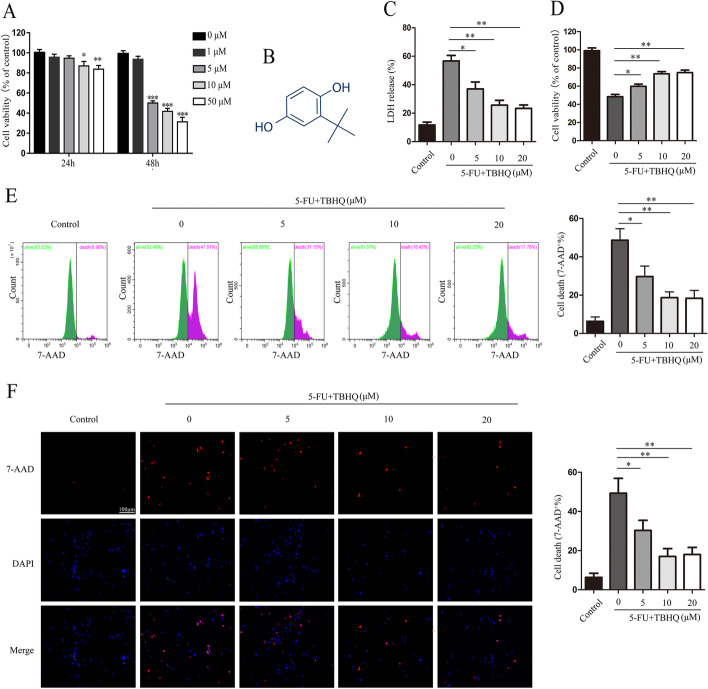
TBHQ alleviates 5-FU-induced intestinal epithelial cell injury. **A** The cytotoxicity of 5-FU was determined using the CCK-8 assay in human intestinal epithelial cells. **B** The chemical structure of TBHQ. **C** 5-FU-induced lactate dehydrogenase (LDH) release was inhibited by treatment with TBHQ. **D** TBHQ increased cell viability following 5-FU treatment in a dose-dependent manner. **E**, **F** 7-AAD-positive cells were stained and counted using flow cytometry and fluorescent microscopy (scale bars: 100 μm). *p < 0.05, **p < 0.01

We first investigated the effect of TBHQ on 5-FU-induced intestinal epithelial cell injury. The CCK-8 assay showed that 5-FU treatment markedly inhibited the proliferative ability of HIECs in a time- and dose-dependent manner (Fig. 1A). Based on these data, we selected a dose of 5 μM and a time point of 48 h for all subsequent cellular experiments.

TBHQ, the structure of which is shown in Fig. 1B, significantly decreased LDH release and improved the proliferative ability of 5-FU-treated HIECs in a dose-dependent manner (Fig. 1C, D). We also found that TBHQ alleviated 5-FU-induced cell death in HIECs (Fig. 1E, F). We further determined the effect of TBHQ treatment alone on intestinal epithelial cells. As expected, TBHQ treatment (10 μM) was not cytotoxic and did not induce intestinal epithelial cell injury (Additional file 1: Fig. S1A–D). These results indicate that 10 μM of TBHQ effectively alleviates 5-FU-induced intestinal epithelial cell injury in vitro.

### TBHQ attenuates 5-FU-induced intestinal mucositis in mice

**Fig. 2:**
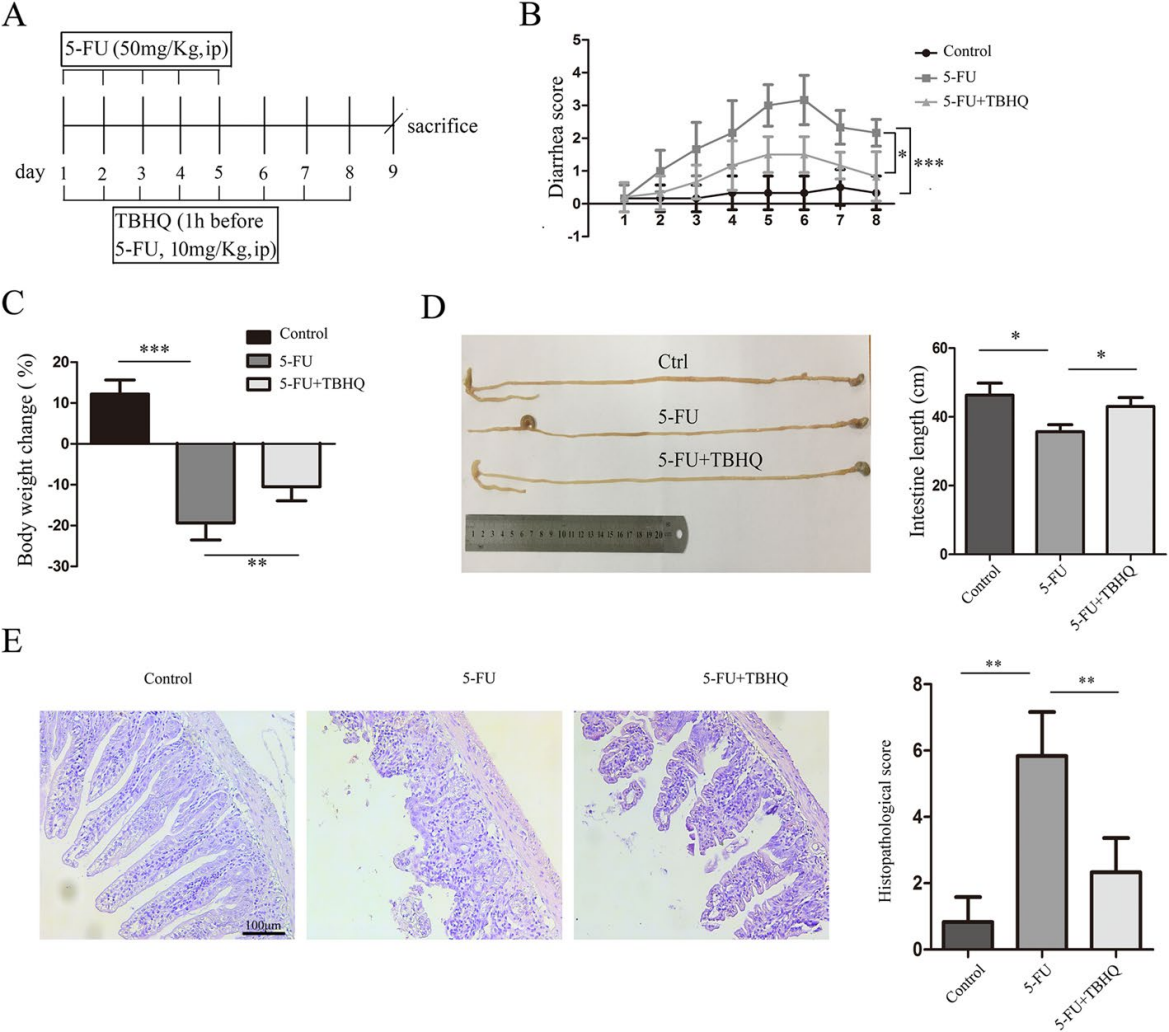
TBHQ attenuates 5-FU-induced intestinal mucositis in mice. **A** Schedule for the administration of TBHQ on 5-FU-induced intestinal mucositis in mice. TBHQ (10 mg/kg body weight in DMSO, i.p.) or vehicle (DMSO, i.p.) was administered from day 1 to day 8, and 5-FU (50 mg/kg body weight, i.p.) or vehicle (saline, i.p.) was administered from day 1 to day 5. Animals were Killed on day 9 (n = 6 animals/group). **B**, **C** The body weight **B** and diarrhea **C** were recorded daily. **D** The whole intestine length of mice in each group was measured. **E** Histological changes in the small intestine were assessed using hematoxylin and eosin (H&E) staining (ccale bars: 100 μm). *p < 0.05, **p < 0.01, ***p < 0.001

Next, we investigated the effect of TBHQ on 5-FU-induced intestinal mucositis in vivo (Fig. 2A). TBHQ significantly attenuated 5-FU-induced diarrhea (Fig. 2B) and loss of body weight (Fig. 2C). Interestingly, the loss of intestinal length induced by 5-FU administration was significantly decreased by TBHQ treatment (Fig. 2D). Administration of TBHQ also relieved mucosal damage and infiltration of inflammatory cells compared to the 5-FU group (Fig. 2E). Furthermore, we determined the effect of TBHQ treatment alone on intestinal mucositis. As shown in Additional file 1: Fig. S1E–K, TBHQ treatment (10 mg/kg body weight) had no cytotoxic effect in vivo and did not induce intestinal mucositis.

### TBHQ ameliorates 5-FU-induced intestinal mucosal barrier destruction

Tight junctions are essential in regulating the intestinal mucosal barrier and controlling the movement of microbes and toxins across the epithelia. We determined the expression of ZO-1, occludin, and claudin-5, which are the protein components of the tight junctions, using western blotting (Additional file 1: Fig. S2A–D), IF (Additional file 1: Fig. S2E), and IHC (Additional file 1: Fig. S2F), and found that their expressions obviously decreased after 5-FU treatment. This effect was significantly reversed by TBHQ.

### *TBHQ inhibits 5-FU-induced inflammatory cytokine expressions and ferroptosis in intestinal epithelial cells *via* the Nrf2/HO-1 pathway*

**Fig. 3:**
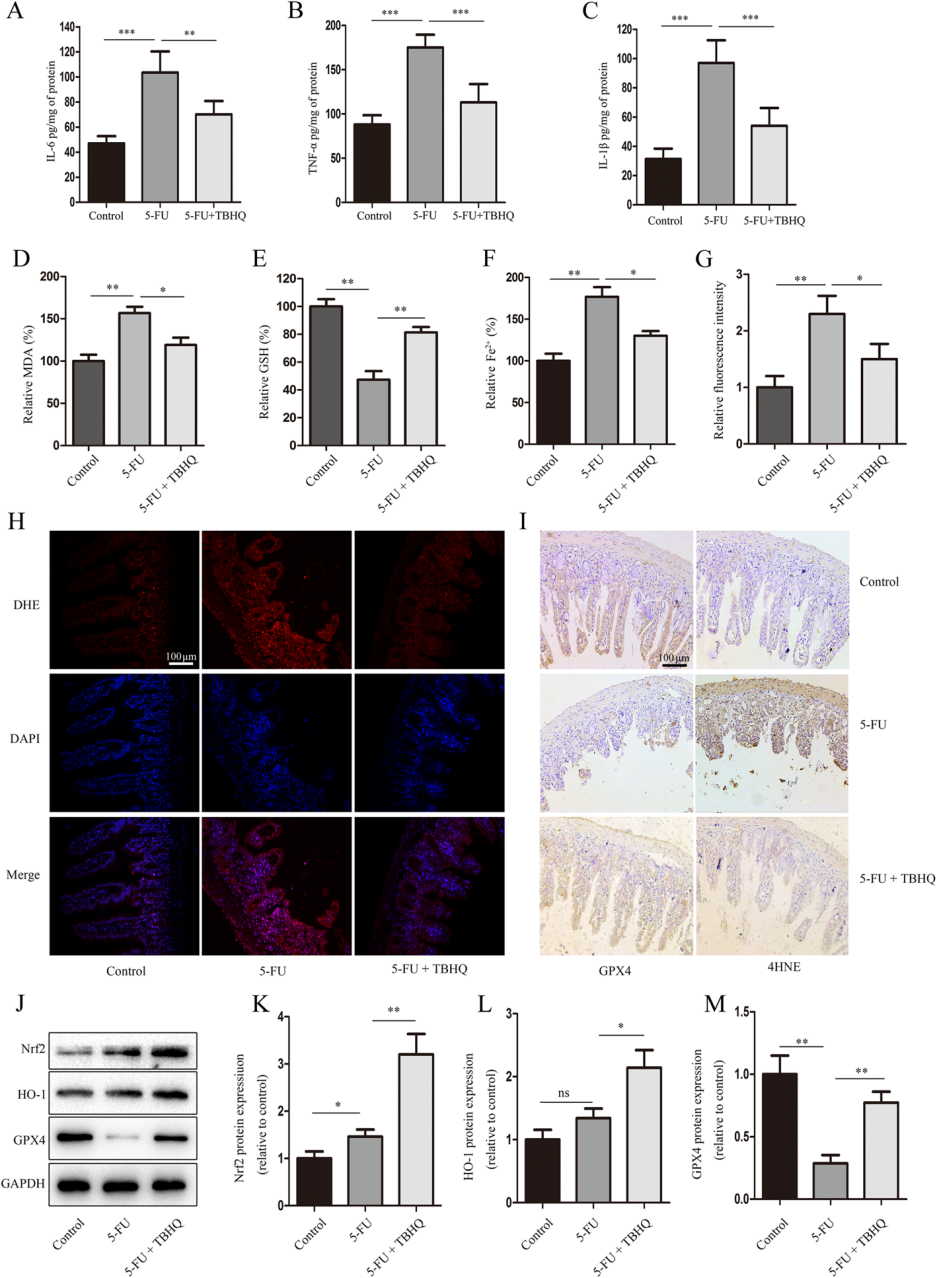
TBHQ inhibits 5-FU-induced intestinal epithelial cell ferroptosis via the Nrf2/HO-1 pathway. A through C – The expressions of IL-6, TNF-α, and IL-1β. D through F –The relative levels of malondialdehyde (MDA), glutathione (GSH), and Fe^2+^. G and H The levels of reactive oxygen species (ROS) were assessed using DHE staining. I – The expressions of 4HNE and GPX4 (scale bars: 100 μm). J through M – The expressions of Nrf2, HO-1, and GPX4 in the small intestine tissues were determined using western blotting. *p < 0.05, **p < 0.01

The levels of IL-1β, IL-6, and TNF-α in the 5-FU-treated intestinal tissue were significantly higher than in the control group (Fig. 3A–C). Interestingly, TBHQ treatment reduced the expression levels of inflammatory cytokines in the intestinal tissue of 5-FU-treated mice (Fig. 3A–C). Chemotherapy drugs usually induce the production of a large amount of ROS, and ROS are the driving forces of ferroptosis.

A previous study showed that 5-FU markedly increases ROS levels in the intestine [22], prompting us to further explore whether ferroptosis is involved in 5-FU-induced intestinal mucositis. To determine whether TBHQ attenuates 5-FU-induced intestinal mucositis by inhibiting ferroptosis, we assessed the indices of ferroptosis, including the products of MDA (Fig. 3D), Fe^2+^ (Fig. 3F), ROS (Fig. 3G, H), 4-HNE (Fig. 3I), and the degradation of GSH (Fig. 3E) and GPX4 (Fig. 3I). As expected, TBHQ significantly attenuated 5-FU-induced ferroptosis in the intestinal epithelial cells.

To investigate whether TBHQ modulates Nrf2/HO-1 signaling in 5-FU-induced intestinal mucositis, the protein expressions of Nrf2 and HO-1 were determined. As shown in Fig. 3J–M, compared to the control group, 5-FU treatment increased the Nrf2 and HO-1 protein levels in vivo. In addition, TBHQ treatment significantly increased Nrf2 and HO-1 expression compared to the 5-FU group. Collectively, these results indicate that TBHQ inhibits 5-FU-induced ferroptosis via the Nrf2/HO-1 pathway.

### Fer-1 alleviates 5-FU-induced intestinal mucositis

**Fig. 4:**
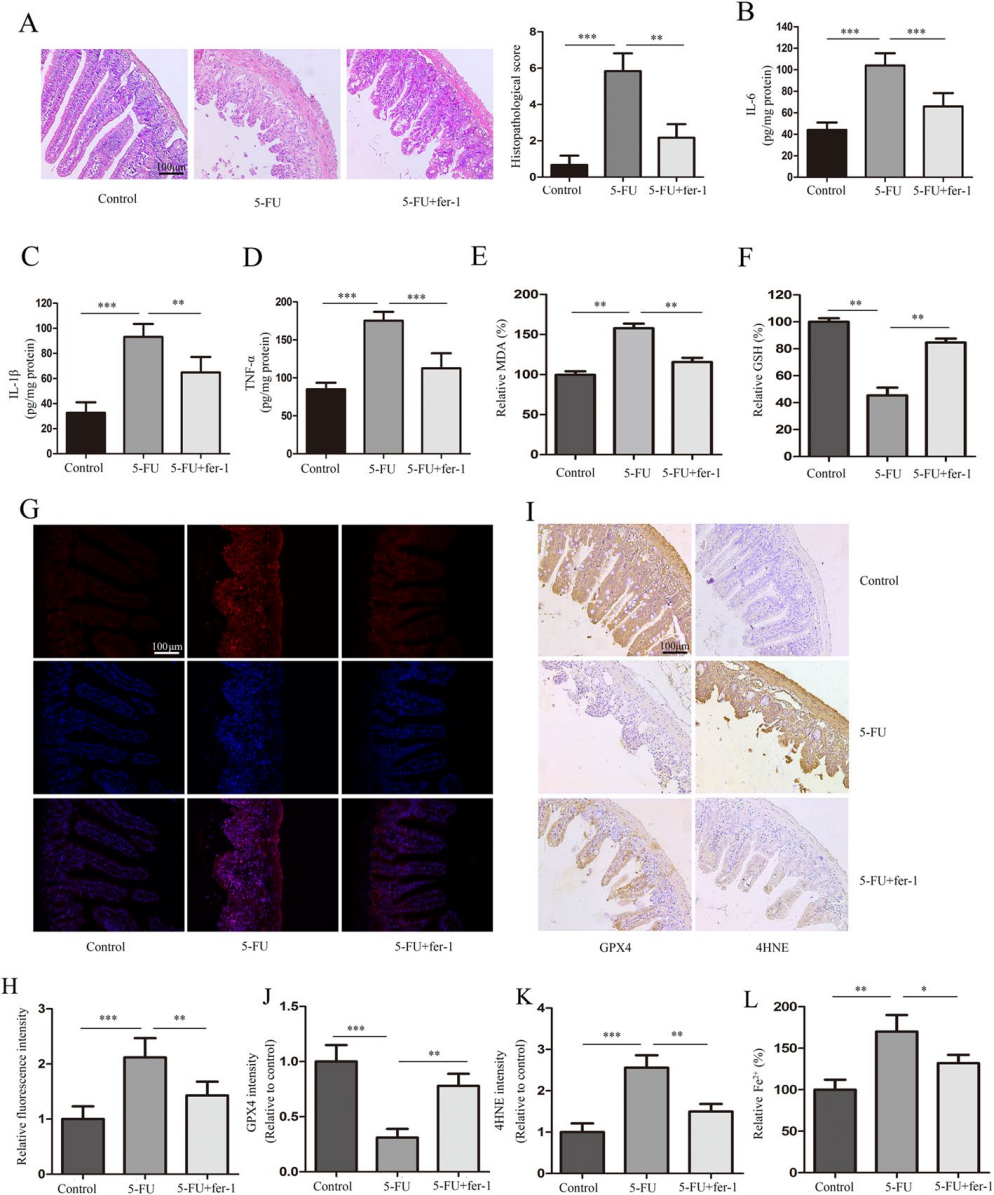
Fer-1 alleviates 5-FU-induced intestinal mucositis. Mice were treated with Fer-1 or DMSO in the presence or absence of 5-FU. **A** Hematoxylin and eosin (H&E) staining and scoring of pathological sections. **B** through **D** The expressions of IL-6, IL-1β, and TNF-α. **E**, **F** The relative levels of malondialdehyde (MDA) and glutathione (GSH) as determined using commercial assay kits. **G**, **H** The levels of reactive oxygen species (ROS) in the intestinal tissues were assessed using DHE staining, and the quantitative results are shown (scale bars: 100 μm). **I** through **K** The expressions of GPX4 and 4HNE determined using immunohistochemistry (scale bars: 100 μm). **L **The relative levels of Fe ^2+^.**p < 0.01, ***p < 0.001

To further elucidate the role of ferroptosis in 5-FU-induced intestinal mucositis, we analyzed the effect of the ferroptosis inhibitor Fer-1 on the condition. Through H&E staining of the pathological sections (Fig. 4A) and the determination of IL-6, IL-1β, and TNF-α levels (Fig. 4B–D), we found that Fer-1 significantly inhibited 5-FU-induced intestinal mucositis. In addition, 5-FU treatment increased the production of lipid peroxide MDA (Fig. 4E), 4-HNE (Fig. 4I, K), ROS (Fig. 4G, H), and Fe^2+^ (Fig. 4L), while decreasing the levels of GSH (Fig. 4F) and GPX4 (Fig. 4I, J), which are characteristic indicators of ferroptosis. By contrast, Fer-1 markedly inhibited 5-FU-induced changes in these indicators. Collectively, these results further indicate that ferroptosis inhibition could alleviate 5-FU-induced intestinal mucositis.

### Nrf2 knockdown reverses TBHQ-induced inhibition of ferroptosis

**Fig. 5:**
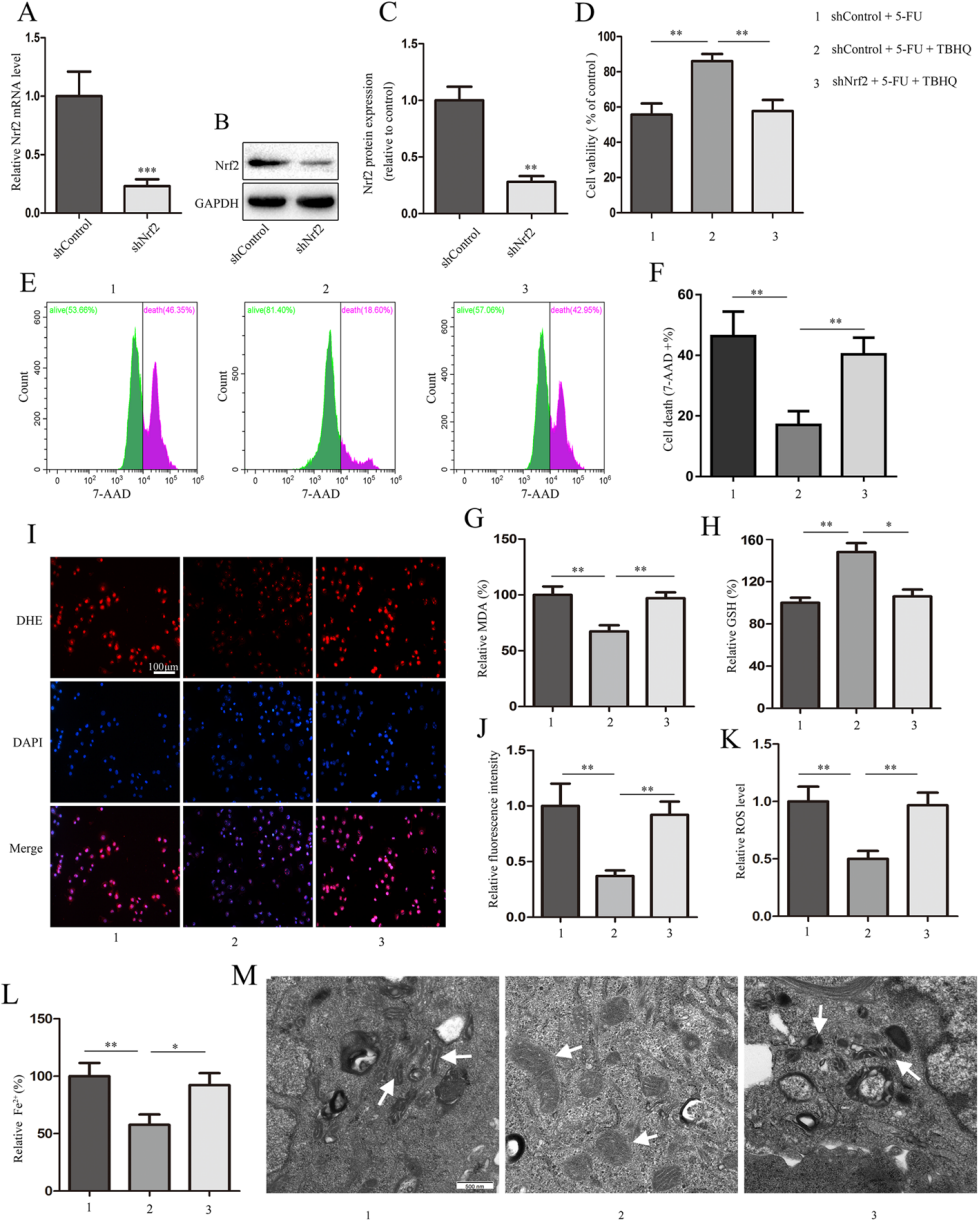
Nrf2 knockdown reverses TBHQ-induced inhibition of ferroptosis. Stable Nrf2-knockdown or control HIECs were treated with TBHQ (10 μM) in the presence of 5-fluorouracil (5-FU; 5 μM) for 48 h. **A** through **C** The expression of Nrf2 determined using quantitative real-time PCR and western blotting assays. **D** through **F** Measurement of cell viability and cell death. **G**, **H** The relative levels of malondialdehyde (MDA) and glutathione (GSH). **I** through **K** The levels of reactive oxygen species (ROS) in HIECs was assessed using DHE staining and flow cytometry, and the quantitative results are shown (scale bars: 100 μm). **L** The relative levels of Fe ^2+^. **M** Transmission electron microscope images of representative mitochondrial structures (indicated by arrows) in HIECs (scale bar, 500 nm). *p < 0.05, **p < 0.01, ***p < 0.001

To further verify the role of Nrf2 in TBHQ-mediated inhibition of ferroptosis, we generated Nrf2-knockdown HIEC lines. Nrf2 knockdown was confirmed using quantitative real-time PCR and western blotting (Fig. 5A–C). Interestingly, Nrf2 knockdown significantly reversed the TBHQ-induced protection of cell viability and inhibition of cell death (Fig. 5D–F). Moreover, as expected, the known ferroptotic events ROS production (Fig. 5I–K), GSH depletion (Fig. 5H), lipid formation (Fig. 5G), shrinking of the mitochondria, Fe^2+^ levels (Fig. 5L), and decrease in the mitochondrial cristae (Fig. 5M) occurred more in Nrf2-silenced cells than in the control cells. These results indicate that TBHQ inhibits 5-FU-induced ferroptosis in intestinal epithelial cells via Nrf2/HO-1 pathway activation.

## Discussion

Intestinal mucositis is one of the most common adverse reactions during chemotherapy. It is an inflammatory or ulcerative disease with pathological features that include atrophy of the intestinal villi and crypt hyperplasia. The primary clinical symptoms are anorexia, nausea, vomiting, diarrhea, and abdominal pain. Patients can also display more serious symptoms, including water electrolyte and acid–base imbalance, malnutrition, secondary bacteremia, infection, sexual shock, and multiple organ dysfunction [23]. 5-FU is a commonly used chemotherapeutic drug for the treatment of digestive system tumors and breast cancer, among others. However, approximately 50–80% of patients treated with 5-FU show intestinal mucosal damage [24].

The pathological mechanism of chemotherapy-induced intestinal mucositis is very complex and has not been fully elucidated. Moreover, there is a lack of effective intervention drugs to treat it. At present, it is believed that chemotherapy drugs induce the production of a large amount of ROS, which leads to the death of the intestinal epithelium, the release of inflammatory factors, and the destruction of the intestinal mucosal barrier, eventually leading to intestinal mucositis [25, 26].

As an activator of Nrf2, TBHQ has been considered to effectively inhibit ROS generation [17–19, 27]. In this study, we explored the role of TBHQ in 5-FU-induced intestinal mucositis and found that TBHQ contributes to a reduction in 5-FU-induced intestinal epithelial cell injury in vitro and intestinal mucositis in vivo.

Studies have shown that the administration of 5-FU to experimental animals induces a decrease in body weight, diarrhea, and a release of inflammatory cytokines, accompanied by morphological damage to the intestine [22, 26]. Therefore, we looked at the influence of TBHQ on body weight, diarrhea, and the release of inflammatory factors in 5-FU-treated mice. As expected, TBHQ significantly decreased the loss of body weight, inflammatory cytokine release, and diarrhea.

Tight junctions are multiple protein complexes that are the main connections between intestinal mucosal epithelial cells. They play an important role in maintaining mechanical integrity and the mucosal barrier. It is well established that the proteins ZO-1, occludin, and claudin-5 play central roles in maintaining the integrity of the tight junctions and the mucosal barrier function [28, 29]. Thus, we determined the expression of these three tight junction proteins and found that TBHQ increased their expression in the intestinal mucosal epithelial cells of 5-FU-treated mice.

Ferroptosis is categorized as regulated necrosis. It has been shown to occur in various intestinal diseases, including intestinal ischemia reperfusion injury, ulcerative colitis, and colorectal cancer. In this study, we showed that ferroptosis is involved in 5-FU-induced intestinal mucositis in vivo and that TBHQ can inhibit 5-FU-induced ferroptosis.

We further investigated the underlying mechanism by which TBHQ functions in 5-FU-induced intestinal mucositis. The Nrf2/HO-1 signaling pathway is considered to be one of the most critical endogenous antioxidative stress pathways and an important target for inflammation-related diseases. Convincing evidence shows that HO-1 is the key protein in the occurrence of ferroptosis, and there are many drugs that inhibit ferroptosis by activating the Nrf2/HO-1 signaling pathway [6, 30, 31]. Our results show that TBHQ inhibits 5-FU-induced ferroptosis via the activation of Nrf2.

## Conclusions

We evaluated the efficacy of TBHQ treatment on 5-FU-induced intestinal epithelial cell injury in vitro and intestinal mucositis in vivo. Our results demonstrate that the administration of TBHQ has significant protective effects against 5-FU-induced damage. Potential mechanisms involve the activation of Nrf2 and attenuation of 5-FU-induced ferroptosis. These findings suggest that TBHQ may be a promising novel therapeutic candidate for the prevention of intestinal mucositis during cancer chemotherapy.

## Supplementary Information

**Additional file 1: Figure S1. **The cytotoxicity of TBHQ in vitro and in vivo. The cytotoxicity of TBHQ was detected by CCK-8 assay (**a**), Lactate dehydrogenase (LDH) release assay (**b**), and 7-AAD staining **c**, **d** in human intestinal epithelial cells (HIECs). The body weight **e** and whole intestine length **f**, **g** of mice in each group was measured. **h** Histological changes in the small intestine were assessed by hematoxylin and eosin staining (Scale bars: 100 μm). The expression of IL-6 (**i**), TNF-α (**j**), and IL-1β (**k**). NS: P>0.05. **Figure S2. **TBHQ ameliorates 5-FU-induced intestinal mucosal barrier destruction. The expression of tight junction proteins ZO-1, occludin, and claudin-5 was detected by western blotting (**a**–**d**), immunofluorescence (**e**), and immunohistochemistry (**f**). Scale bars: 100 μm. *P<0.05, **P<0.01.

## Data Availability

Not applicable.

## Abbreviations

5-FU: 5-Fluorouracil
Fer-1: Ferrostatin-1
Nrf2: Nuclear factor erythroid 2-related factor 2
TBHQ: Tertiary butylhydroquinone
HIEC: Human intestinal epithelial cell
CCK-8: Cell Counting Kit-8
LDH: Lactate dehydrogenase
i.p.: Intraperitoneally
IHC: Immunohistochemical analysis
IF: Immunofluorescence
GSH: Glutathione
MDA: Malondialdehyde
ROS: Reactive oxygen species
DHE: Dihydroethidium
