# Supplementary material for: TBHQ attenuates ferroptosis against 5-fluorouracil-induced intestinal epithelial cell injury and intestinal mucositis via activation of Nrf2
Source: Cell Mol Biol Lett. 2021 Nov 18;26:48. doi: 10.1186/s11658-021-00294-5 (PMC8600870; doi:10.1186/s11658-021-00294-5)

**Figure S1.** The cytotoxicity of TBHQ in vitro and in vivo. The cytotoxicity of TBHQ was detected by CCK-8 assay (a), Lactate dehydrogenase (LDH) release assay (b), and 7-AAD staining (c, d) in human intestinal epithelial cells (HIECs). The body weight (e) and whole intestine length (f, g) of mice in each group was measured. (h) Histological changes in the small intestine were assessed by hematoxylin and eosin staining (Scale bars: 100 μm). The expression of IL-6 (i), TNF-α (j), and IL-1β (k). NS: P > 0.05


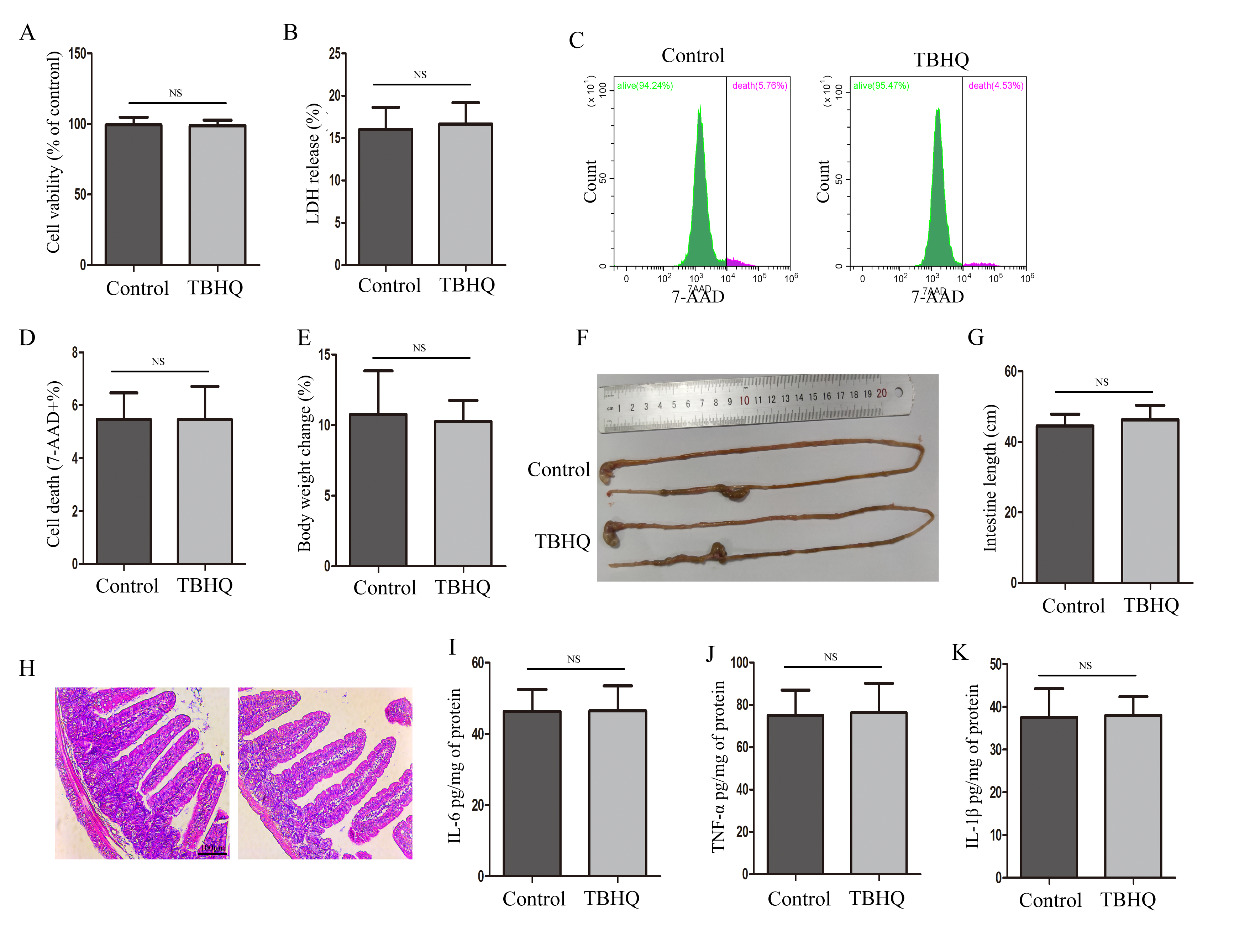


**Figure S2.** TBHQ ameliorates 5-FU-induced intestinal mucosal barrier destruction. The expression of tight junction proteins ZO-1, occludin, and claudin-5 was detected by western blotting (a–d), immunofluorescence (e), and immunohistochemistry (f). Scale bars: 100 μm. *P < 0.05, **P < 0.01.


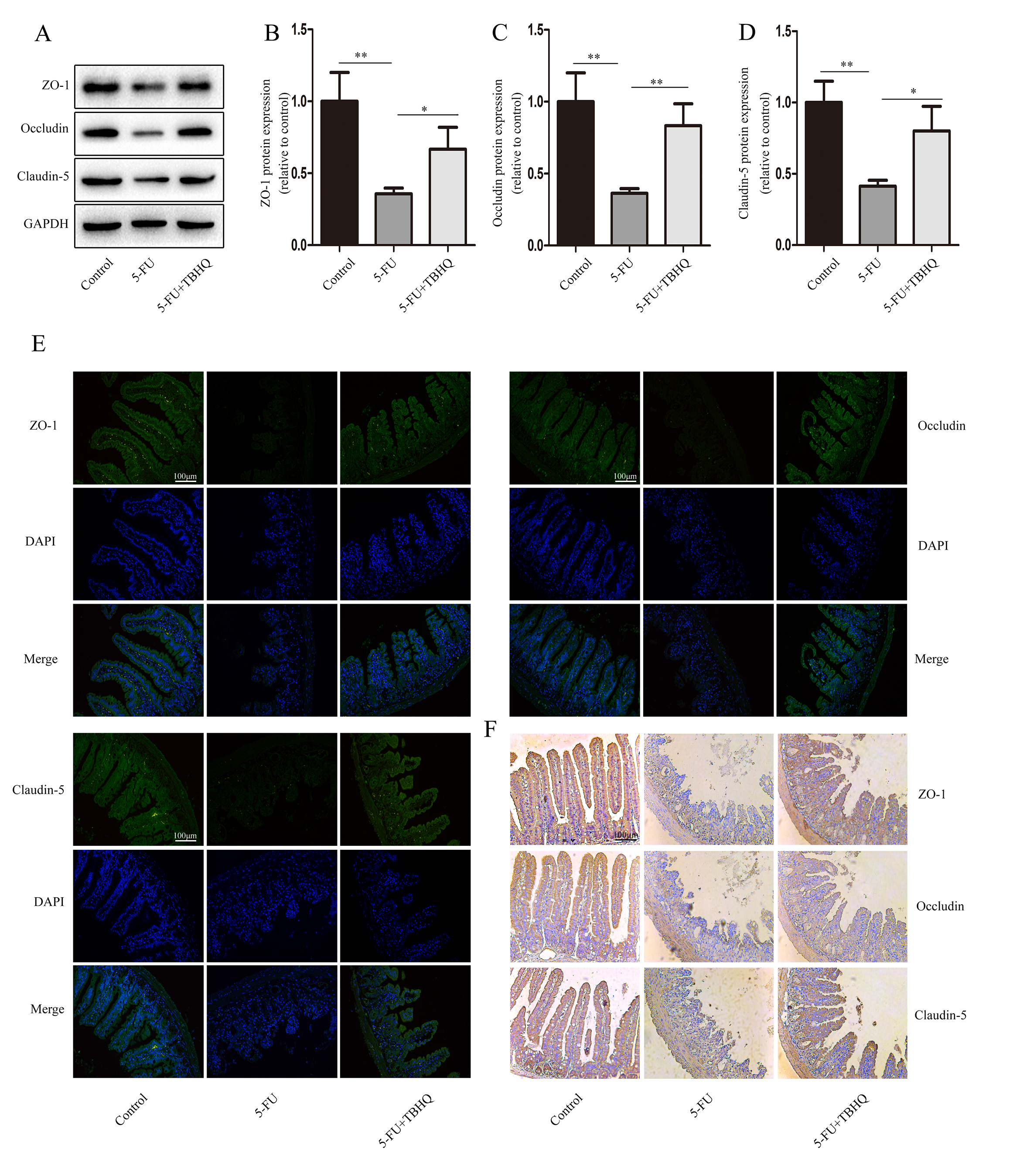

Supplement: Supplementary file 1 — Additional file 1: Figure S1. The cytotoxicity of TBHQ in vitro and in vivo. The cytotoxicity of TBHQ was detected by CCK-8 assay (a), Lactate dehydrogenase (LDH) release assay (b), and 7-AAD staining c, d in human intestinal epithelial cells (HIECs). The body weight e and whole intestine length f, g of mice in each group was measured. h Histological changes in the small intestine were assessed by hematoxylin and eosin staining (Scale bars: 100 μm). The expression of IL-6 (i), TNF-α (j), and IL-1β (k). NS: P>0.05. Figure S2. TBHQ ameliorates 5-FU-induced intestinal mucosal barrier destruction. The expression of tight junction proteins ZO-1, occludin, and claudin-5 was detected by western blotting (a–d), immunofluorescence (e), and immunohistochemistry (f). Scale bars: 100 μm. *P<0.05, **P<0.01. [file 11658_2021_294_MOESM1_ESM.doc]
